# Virtual surgical planning/3D printing assisted fibula osteoseptocutaneous flap combined with anterolateral thigh flaps for extensive composite oromandibular defects reconstruction: a retrospective study of case series

**DOI:** 10.3389/fbioe.2023.1273318

**Published:** 2023-11-02

**Authors:** Yaoxiang Xu, Yali Li, Wenlin Xiao, Jin Yue, Lingfa Xue, Li Li, Zexian Xu, Jian Sun

**Affiliations:** ^1^ Department of Oral and Maxillofacial Surgery, The Affiliated Hospital of Qingdao University, Qingdao, China; ^2^ School of Stomatology, Qingdao University, Qingdao, China; ^3^ Dental Digital Medicine and 3D Printing Engineering Laboratory of Qingdao, Qingdao, China; ^4^ Shandong Provincial Key Laboratory of Digital Medicine and Computer-Assisted Surgery, Qingdao, China

**Keywords:** flap transplantation, reconstructive surgery, virtual surgical planning, multipleflap, 3D printing

## Abstract

Oromandibular tumors or osteoradionecrosis often lead to extensive composite defects encompassing intraoral, bone and extraoral tissues. A single flap cannot simultaneously offer sufficient bone and soft tissue. The combination of free flaps could be a prospective approach to overcome the challenge. The study aims to assess the efficacy of virtual surgical planning (VSP) and 3D printing assisted fibula osteoseptocutaneous flap (FOSCF) combined with anterolateral thigh flaps (ALT) in reconstructing extensive composite defects in the oromandibular region. A retrospective analysis was conducted on 8 patients who underwent reconstruction using FOSCFs combined with ALTs. Post-surgical excision of the lesions, we obtained mean values for the defects of intraoral soft tissue, bone, extraoral soft tissue, namely, being 42.7 cm^2^, 96 mm, and 68.9 cm^2^. The mean surgical procedures took 712.5 min. A total of 16 flaps were harvested and transplanted for the 8 patients, with all successfully surviving. Postoperatively, complications manifested as localized intraoral infections in 2 cases, intermuscular vein thrombosis in another 2 cases, and pulmonary infections in 2 patients. Two patients unfortunately experienced tumor recurrence, at 12 and 3 months post-operation respectively. For the surviving 6 patients, the average follow-up period was 12.2 months. Regarding patient satisfaction, one expressed dissatisfaction with the contour of the mandible, and two exhibited moderate trismus. Objective assessments identified 1 case of oral incontinence and 2 cases where external flap contractures were observed. All 8 patients experienced restoration of masticatory function and were able to consume a soft diet within a month post-surgery. VSP/3D printing assisted FOSCFs combined with ALTs can be performed safely to reconstruct the extensive composite tissue defects in our study, with desirable esthetic and functional results, and it is a reliable option in selecting patients with defects involving multiple tissue types. However, the benefits of this method needed more cases to validate.

## 1 Introduction

Extensive composite oromandibular defects are frequently caused by surgical resection of sizeable primary tumors in the head and neck region, or the result of osteoradionecrosis (ORN) post-radiotherapy. The reconstruction of such complex defects, encompassing multiple structures such as external skin, mandible, and oral mucosa presents a formidable challenge. Free flap reconstruction is the standard of care in these extensive defects, while there may not exist a single free flap capable of simultaneously offering sufficient bone stock and soft tissue. In these instances, the combination of multiple free flaps proves to be an efficacious treatment strategy (Lee et al., 2010; Weitz et al., 2015).

Selecting suitable free flap is integral to the reconstructive success and overall outcome. In contrast to iliac or scapular osteocutaneous free flaps, the FOSCF can provide longer bone segments as well as ample skin paddle for intraoral lining (Taylor et al., 2016; Liu et al., 2022). The ALT is the most useful workhorse flap employed for microsurgical reconstruction. Depending on requirements, the flap can be harvested as myocutaneous, fasciocutaneous, adipofascial, or in combination with the adjacent tissues or muscles as chimeric flaps. This versatility makes it particularly suited for the reconstruction of extensive extraoral skin defects (Hsieh et al., 2021; Ranganath et al., 2022). The advent of VSP and 3D printing marks a significant milestone for the FOSCF. The utilization of these techniques enhances the safety, accuracy, and symmetry of this surgical procedure while considerably reducing the operating time (Ritschl et al., 2021; Al-Sabahi et al., 2022; Idris et al., 2022).

Despite their advantages, multi-flap approaches for the reconstruction of composite oromandibular defects continue to be challenging and are a subject of ongoing debate. A few reports are available on the functional and aesthetic outcome of double free flap reconstructions in these defects (Lee et al., 2010; Tharakan et al., 2023). The objective of this study is to evaluate the effect of the combination of VSP/3D printing-assisted FOSCF with ALT in composite tissue defects reconstruction which could not be solved by one single flap.

## 2 Patients and methods

This study received approval from the Ethics Committee at the Affiliated Hospital of Qingdao University, Qingdao (ethics number, QYFYWZLL 27958). A retrospective review was conducted of 8 patients who underwent extensive composite oromandibular defects repair using the combination of VSP/3D printing-assisted FOSCF with ALT between July 2019 and December 2022 at our department.

The cohort comprised seven males and one female. All patients had undergone lesion resection, with extensive defects variably involving oral mucosa, mandible, and perioral skin areas. All defects were reconstructed using a FOSCF in combination with ALT. A two-team approach was consistently employed throughout all stages of the operation.

### 2.1 Preparation of the FOSCF assisted by VSP and 3D printing

All patients underwent routine preoperative three-dimensional CT scan of the mandible and fibula (SOMATOM Force CT, slice thickness 0.625 mm), in addition to lower limb CT angiography (CTA). The acquired data were input into the Mimics 17.0 software (Materialise, Leuven, Belgium) in the DICOM format. The software was used to simulate tumor excision, fibula osteotomy, mandibular reconstruction, and the design of repositioning guides. In the process, the mandibular osteotomy guide and the fibula positioning guide share the same nail track to ensure the accuracy of mandibular reconstruction. For those mandibular defects not traversing the midline, mirror technology was employed to accomplish reconstruction. However, in cases where the defects did cross the midline, a synergistic approach combining mirror and surface reconstruction techniques was adopted. Following the completion of the virtual surgery, the designed guides were materialized using an 3D printer (UltraCraft A2D, HeyGears), and subsequently sterilized with plasma. The reconstruction-team prepared the FOSCF. The skin perforator was located and the skin paddle was incised in accordance with the magnitude of the intraoral soft tissue defect. The fibula osteotomy guide was positioned, leading to the fibula being cut to the length prescribed in the virtual surgery. The distal end of the fibular artery was ligated, preserving the proximal vascular pedicle. The fibula was then contoured using the shaping guide and secured in position with miniplates. Upon completing the FOSCF preparation, the same team proceeded to harvest the ALT.

### 2.2 Oral lesion excision

The resection-team was tasked with the excision of the tumor at its safe boundary. According to the preoperative virtual surgical design, the mandibular osteotomy guide was positioned, followed by the segmental resection of the mandible. After the complete excision of the tumor, two sets of recipient vessels were prepared. Subsequent to the surgical procedure, patients were admitted to the intensive care unit (ICU), and tracheostomy was not a routine intervention. Patients were typically transferred back to the general ward within a period of 3–5 days postoperatively, contingent upon their stabilized condition. All patients needed nasogastric feeding for 1 week post-surgery. Subsequently, a soft or normal diet was gradually adopted. All patients except No.5 and No.8 underwent postoperative radiation therapy.

Surgical and medical complications were documented. A patients satisfaction questionnaire was administered to make subjective evaluation on chewing, speech, dry mouth and facial appearance, which were divided into three grades: very satisfied, satisfied and dissatisfied. To determine objective evaluation, facial appearance, oral incontinence, speech problems, eating problems, xerostomia, and flap contracture were assessed. Each individual item was scored using a Likert scale (1 = extremely abnormal and 5 = completely normal).

## 3 Results

Our study comprised 8 patients including 7 males and 1 female, aged from 45 to 68 years (mean, 60.5 years). Seven of these patients presented with oral malignant tumors, all diagnosed as squamous cell carcinoma, while one patient (No.5) had osteoradionecrosis as a consequence of radiation therapy. A total of 16 flaps were prepared for 8 patients, which consisted 8 FOSCFs and 8 ALTs. Post-excision of the lesions, the dimensions of the intraoral soft tissue defects ranged from 7.5 × 4 cm to 12 × 6.5 cm (mean, 42.7 cm^2^). The length of the bone defects extended from 76 mm to 125 mm (mean, 96 mm), while the extraoral soft tissue defects varied from 8 × 5 cm to 14 × 9 cm (mean, 68.9 cm^2^). The anastomosis involved recipient arteries that included the facial artery, superior thyroid artery, and lingual artery. Concurrently, the recipient veins involved were the facial vein, superior thyroid vein, internal jugular vein, and external jugular vein. One case required vascular grafting. The operative duration for these procedures ranged between 600 and 840 min, with an average of approximately 712.5 min (Table 1).

**TABLE 1:** Patient data.

Case no.	Age (Y)	Sex	Tumor	Location	Stage	ILD (cm)	OLD (cm)	BD (mm)	Arteries used	Veins used	Operation	Survived/Deceased
											Time (min)	
1	61	M	SCC	Buccal	T4N0M0	9 × 5	11 × 6	100	Facial; superior thyroid	External jugular; facial	720	Deceased (12 months, recurrence)
2	68	F	SCC	Buccal	T4N1M0	7 × 5.5	10 × 6.5	109	Facial; Contralateral facial	Facial; Contralateral facial	780	Deceased (3 months, recurrence)
3	66	M	SCC	Buccal	T4N0M0	7.5 × 4	8 × 5	76	Facial; superior thyroid	External jugular; facial	660	Survived (18 months)
4	65	M	SCC	Gum	T4N0M0	9 × 4.5	12 × 6.5	108	Facial; Lingual	Facial; internal jugular	710	Survived (15 months)
5	70	M	ORN	Buccal	/	7 × 4.5	9 × 6	78	Facial; superior thyroid	Facial; internal jugular	730	Survived (12 months)
6	48	M	SCC	Buccal	T4N0M0	8.5 × 5.5	10 × 8	80	Facial; superior thyroid	Facial; internal jugular	660	Survived (11 months)
7	45	M	SCC	Buccal	T4N1M0	12 × 6.5	14 × 9	125	Facial; superior thyroid	External jugular; internal jugular	840	Survived (10 months)
8	61	M	SCC	Mouth floor	T4N0M0	7 × 4.5	8.5 × 5	92	Facial; superior thyroid	Facial; superior thyroid	600	Survived (7 months)

ILD, inner lining defect; BD, bone defect; OLD, outer lining defect; SCC, squamous cell carcinoma; ORN, osteoradionecrosis.

Remarkably, all of the flaps survived without any vascular crises or local flap necrosis. Two cases (No.3, No.4) developed local oral infections. Additionally, intermuscular vein thrombosis in the lower limbs was observed 2 patients (No.2, No.5). Pulmonary infection was found in 2 cases (No.2, No.4). Unfortunately, 2 of the 8 patients succumbed due to tumor recurrence at 12 months and 3 months post-surgery respectively. The mean follow-up time of the remaining 6 patients was 12.2 months (range, 7 months–18 months). Patient satisfaction assessments revealed one individual (No.3) was dissatisfied with their post-operative appearance, while two (No.2, No.4) reported moderate trismus. However, the remaining patients all reported satisfaction levels above average. Objective evaluations indicated oral incontinence in 1 case (No.4) and external flap contracture in 2 cases (No.2, No.4). Impressively, all 8 patients regained masticatory function and resumed a soft diet within a month post-operation.

### 3.1 Case presentation

**FIGURE 1:**
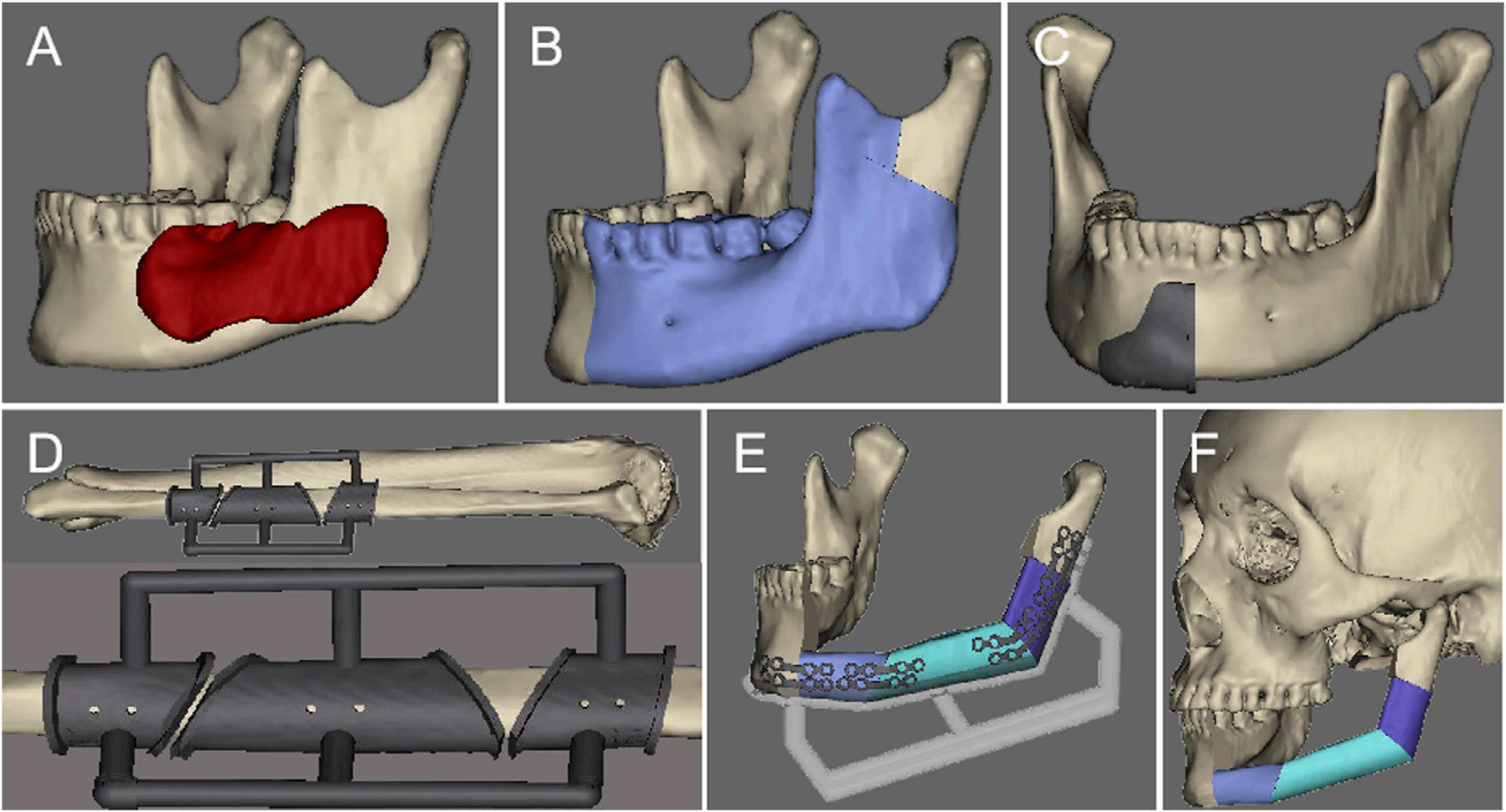
The preoperative virtual surgical design: **(A)**. Determine the range of the lesion. **(B)** Simulate the range of bone cutting. **(C)** Design the bone cutting guide. **(D)** Design the fibula osteotomy guide. **(E)** Design the fibula repositioning guide. **(F)** Restore the mandibular contour.

**FIGURE 2:**
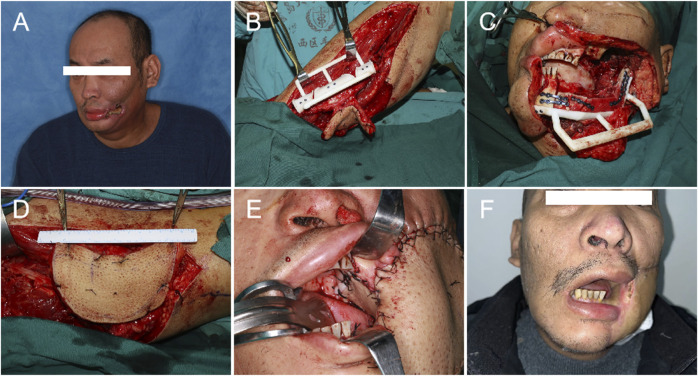
Operational procedure: **(A)**. Preoperative photo. **(B)** Fibula shaping. **(C)** Fibula positioning. **(D)** Harvest of the ALT. **(E)** The fibula osteoseptocutaneous flap was used to repair the oral soft tissue and the mandible, and the ALT was used to repair the extraoral tissues. **(F)** 6 months post-operation.

A 45-year-old male patient diagnosed with a squamous cell carcinoma on his left cheek. The tumor demonstrated regression by radiation therapy (66 Gy) 7 months ago. However, the anterior part was ulcerated and gradually formed a penetrating defect after 1 month. Histopathological examination confirmed squamous cell carcinoma, staged clinically as T4N1M0. With the assistance of VSP and 3D printing technology, the reconstruction utilising both flaps was successfully performed (Figures 1, 2).

## 4 Discussion

Surgical removal of oral tumors or osteoradionecrosis can result in substantial composite tissue defects, presenting formidable challenges for reconstructive repair. A single flap is often insufficient to adequately address these extensive defects. Consequently, the combination of multiple flaps has emerged as an innovative therapeutic approach (Lee et al., 2010; Weitz et al., 2015; Hsieh and Bewley, 2019; Silva et al., 2019; Moratin et al., 2021; Raghuram et al., 2021; Santilli et al., 2021; Tharakan et al., 2023). The ALT can furnish sufficient tissue volume and can be fashioned into a chimeric flap with minimal impact on the donor site, rendering it an optimal choice for repair and reconstruction of large soft tissue defects in the oromandibular region (Thomas et al., 2020). The FOSCF is exceptionally suitable for large bone tissue defects. Its accompanying skin paddle can repair tissues both inside or outside the oral cavity, and its associated muscle can effectively fill the surgical dead space (Dowthwaite et al., 2013). Therefore, for large composite tissue defects in the oromandibular region, we opt for the ALT for the repair of extraoral soft tissue defects and the FOSCF for the repair of bone and intraoral soft tissue defects. Nevertheless, surgical alternatives should be carefully considered. The fibular flap can be substituted with a reconstruction plate, iliac bone muscle flap, or scapular bone muscle flap. Soft tissue flap can be replaced with a vascularized free flap or pedicled flap, such as the forearm flap, rectus abdominis flap, or pectoralis major myocutaneous flap. However, the radial forearm flap offers limited soft tissue volume, it is difficult to fill the dead cavity effectively. Its application is further limited by the severe scarring left on the forearm, tendon exposure, and forearm mobility disorders (Ranganath et al., 2022). The rectus abdominis myocutaneous flap can deliver a satisfactory volume of soft tissue, yet it may trigger complications such as reduced abdominal wall strength and incisional hernia. While the pectoralis major flap supplies generous soft tissue, it’s typically considered a backup option. The free iliac flap has insufficient bone length to adequately repair the defects. Besides, the free scapular flap cannot effectively mimic the shape of the mandibula, and has limitations in deficient bone length (Liu et al., 2022). Moreover, reconstruction plates are prone to complications like screw loosening, plate fracture, plate exposure and stress shielding.

A unique aspect of our study is the incorporation of VSP and 3D printing. VSP allows for tailoring surgical approaches, ensuring precise lesion delineation, osteotomy localization, and fibula harvest estimation. Preoperative mandibular modeling not only reduces surgical time but also ensures superior functional and aesthetic outcomes. 3D-printed guides, enhance surgical accuracy, fostering a closer match to the original mandibular structure. Shared screw tracks between the mandibular osteotomy and fibula positioning guides simplify the procedure, further minimizing trauma and operative time (Seruya et al., 2013; Schepers et al., 2015; Wu et al., 2021). Using the osteotomy guide and positioning guide, the fibula is contoured and then affixed with mini titanium plates. Shaw et al. (2004) proved that there were no significant differences in complications between miniplates and reconstruction plates, but the author also highlighted the defects of reconstruction plates, including stress shielding, interference with the vascular pedicle, and problems of metal fatigue when bending plates in the sagittal plane. In comparison, miniplates which avoid the above drawbacks are a better choice. Intermaxillary fixation was performed after surgery for 1 week, encouraging oral exercises to prevent trismus.

The vascular pedicle is severed after the recipient vessels are prepared, markedly reducing ischemic time and overall duration of the surgery. Literature indicates that when the ALT donor site width is under 8.0 cm, primary closure is feasible; for wider defects, skin grafting becomes necessary (Chen and Tang, 2003). Some reports suggest that direct suturing is feasible when the width is less than 10.0 cm (Townley et al., 2011). Studies have proposed that the ratio of flap width to thigh circumference can serve as a reliable metric for direct wound closure, with a ratio less than 16% indicating direct closure (Boca et al., 2010). In our study, we found that for one patient, the flap width was 9.0 cm, and direct suturing did not induce fascial compartment syndrome or other complications.

While the use of double flaps allows for the repair of large composite tissue defects in the oromandibular region, overcoming the limitations of a single flap or composite flap due to vascular pedicle restrictions, it demands higher provisions for neck vessels. This includes the preparation of two sets of anastomotic arteries and veins in the recipient area. In our study, it was found that arteries were relatively easier to prepare, with a total of 16 arteries prepared. The most frequently used anastomotic arteries were the facial artery and the superior thyroid artery, while the facial vein and internal jugular vein were the most commonly used anastomotic veins. Flow-through flaps, as reported in literature (Qing et al., 2015; He et al., 2021), allow the usage of only one set of recipient area vessels. However, this approach carries a significant drawback: if a vascular crisis arises, both flaps are in danger of necrosis. Therefore, we advocate for the preparation of two sets of anastomotic arteries and veins in the recipient area to mitigate such circumstances. Literature suggests that the ligated internal jugular vein can be utilized for end-to-side anastomosis, offering a method for venous anastomosis (Akazawa et al., 2019). Furthermore, it has been reported that the transverse cervical vein and superficial temporal vein can also serve as recipient area vessels (Hansen et al., 2007; Tessler et al., 2017; Wang et al., 2021).

This method affords greater flexibility and autonomy, but it involves extended operation time, high costs, considerable trauma, and numerous potential complications. It mandates superior surgical skill, restricting its broader implementation. Inexperienced medical institutions and young physicians should first master single flap reconstruction before considering advanced techniques. Without this foundation, there’s a risk of increased surgical complications and irreversible outcomes. Additionally, the inclusion of 3D printing escalates treatment costs, making this approach unfeasible for institutions lacking this technology. In our study, though every flap transplantation was triumphant, five cases did confront complications. Two cases suffered from local oral infections and gradually recovered after conservative treatments. Two patients presented with intermuscular vein thrombosis in the lower limbs, which were successfully managed with oral medication, preventing the progression to potential organ embolisms such as cardiac, pulmonary, or cerebral embolism. Additionally, two cases demonstrated a pulmonary infection, which was effectively controlled with antibiotic therapy (Tharakan et al., 2023). reported postoperative complications in multi-flap surgeries to range from 26% to 50%, aligning with our findings. Earlier research indicated a 5-year survival rate for advanced head and neck cancers between 25% and 56% (Abdelmeguid et al., 2021; Mody et al., 2021). Our study, with 2 out of 8 patients succumbing to postoperative tumor recurrence, indicates a marginally superior survival rate, albeit potentially influenced by the relatively shorter follow-up duration. In summary, our study’s outcomes resonate with existing literature, suggesting that this approach doesn’t elevate complications or mortality rates.

It is crucial to acknowledge that patients who are candidates for this procedure are typically in the advanced stages of oral cancer. As such, it is of utmost importance to strictly adhere to the surgical indications:

1) The presence of extensive composite tissue defects, including intraoral, mandibula, and extraoral tissue defects that are too extensive to be repaired with a single flap;
2) The patient must be in good physical health, without obvious surgical contraindications such as congestive heart failure, pulmonary dysfunction, or neck vessel thrombosis;
3) The tumor can be completely resectable without systemic metastasis of lung and bone tissues;
4) The patient must possess a strong desire to combat the disease, as a positive attitude towards treatment is crucial.

However, this study does have limitations. Denture restoration is crucial for patients’ masticatory function (Sozzi et al., 2017). Found that implant survival was high and implant-supported prostheses were a reliable rehabilitation option in patients whose jaws have been reconstruction with fibula-free flap. In our surgical method, fibula was used to prepare for implant repair in the future. However, due to postoperative adjuvant radiotherapy, patients paid more attention to tumor treatment and neglected denture repair. Both function and aesthetics are critically important, yet there is a potential gap in patients’ understanding of these aspects. Consequently, it’s our responsibility to educate them, promoting the benefits of implant restorations to restore masticatory function. Because of the rarity of such patients, we were unable to conduct controlled studies to objectively evaluate the advantages and disadvantages of this method compared to other treatment modalities. Nonetheless, we are confident that as the number of cases increases, the effectiveness of this treatment strategy will be confirmed. Moreover, the success of the double-flap surgery heavily relies on a precise preoperative surgical design and thorough considerations. The surgical approach offers limited flexibility, and the surgical procedure cannot be changed arbitrarily during the operation, as any deviations may result in a mismatch with the original design. Should unforeseen circumstances arise intraoperatively, we are prepared to implement alternative surgical strategies. Developing methodologies to anticipate, mitigate, and adeptly respond to such occurrences will constitute a primary objective in our ongoing research endeavors.

## 5 Conclusion

In conclusion, VSP/3D printing assisted FOSCFs combined with ALTs offers a safe and effective avenue for reconstructing oromandibular massive composite tissue defects in the study. However, the broader benefits and efficacy of this technique necessitate further validation through an expanded patient cohort.

## Data Availability

The original contributions presented in the study are included in the article/supplementary material, further inquiries can be directed to the corresponding author.

## References

[B1] Abdelmeguid A. S. Silver N. L. Boonsripitayanon M. Glisson B. S. Ferrarotto R. Gunn G. B. (2021). Role of induction chemotherapy for oral cavity squamous cell carcinoma. Cancer 127, 3107–3112. 10.1002/cncr.33616 33909292

[B2] Akazawa T. Sekido M. Adachi K. Sasaki K. Aihara Y. Shibuya Y. (2019). End-to-Side venous anastomosis to a ligated vein stump for free flap transfer in head and neck reconstruction. Ann. Plast. Surg. 83, 180–182. 10.1097/sap.0000000000001905 31232824

[B3] Al-Sabahi M. E. Jamali O. M. Shindy M. I. Moussa B. G. Amin A. A. Zedan M. H. (2022). Aesthetic reconstruction of onco-surgical mandibular defects using free fibular flap with and without CAD/CAM customized osteotomy guide: a randomized controlled clinical trial. BMC Cancer 22, 1252. 10.1186/s12885-022-10322-y 36460978 PMC9717507

[B4] Boca R. Kuo Y. R. Hsieh C. H. Huang E. Y. Jeng S. F. (2010). A reliable parameter for primary closure of the free anterolateral thigh flap donor site. Plast. Reconstr. Surg. 126, 1558–1562. 10.1097/prs.0b013e3181ef8cb7 21042113

[B5] Chen H. C. Tang Y. B. (2003). Anterolateral thigh flap: an ideal soft tissue flap. Clin. Plast. Surg. 30, 383–401. 10.1016/s0094-1298(03)00040-3 12916595

[B6] Dowthwaite S. A. Theurer J. Belzile M. Fung K. Franklin J. Nichols A. (2013). Comparison of fibular and scapular osseous free flaps for oromandibular reconstruction: a patient-centered approach to flap selection. JAMA Otolaryngol. Head. Neck Surg. 139, 285–292. 10.1001/jamaoto.2013.1802 23657276

[B7] Hansen S. L. Foster R. D. Dosanjh A. S. Mathes S. J. Hoffman W. Y. Leon P. (2007). Superficial temporal artery and vein as recipient vessels for facial and scalp microsurgical reconstruction. Plast. Reconstr. Surg. 120, 1879–1884. 10.1097/01.prs.0000287273.48145.bd 18090750

[B8] He J. Qing L. Wu P. Zhou Z. Yu F. Tang J. (2021). Large wounds reconstruction of the lower extremity with combined latissimus dorsi musculocutaneous flap and flow-through anterolateral thigh perforator flap transfer. Microsurgery 41, 533–542. 10.1002/micr.30754 33988868

[B9] Hsieh F. Leow O. Q. Y. Cheong C. F. Hung S. Y. Tsao C. K. (2021). Musculoseptocutaneous perforator of anterolateral thigh flap: a clinical study. Plast. Reconstr. Surg. 147, 103e–110e. 10.1097/prs.0000000000007471 33370066

[B10] Hsieh T. Y. Bewley A. (2019). Use of multiple free flaps in head and neck reconstruction. Curr. Opin. Otolaryngol. Head. Neck Surg. 27, 392–400. 10.1097/moo.0000000000000574 31389852

[B11] Idris S. Logan H. Tabet P. Osswald M. Nayar S. Seikaly H. (2022). The accuracy of 3D surgical design and simulation in prefabricated fibula free flaps for jaw reconstruction. J. Pers. Med. 12, 1766. 10.3390/jpm12111766 36579487 PMC9698275

[B12] Lee J. T. Hsu H. Wang C. H. Cheng L. F. Sun T. B. Huang C. C. (2010). Reconstruction of extensive composite oromandibular defects with simultaneous free anterolateral thigh fasciocutaneous and fibular osteocutaneous flaps. J. Reconstr. Microsurg 26, 145–151. 10.1055/s-0029-1242134 19902408

[B13] Liu A. Q. Deane E. C. Heffernan A. Ji Y. Durham J. S. Prisman E. (2022). Patient-reported outcomes and morbidity after head and neck reconstructions: an evaluation of fibular and scapular free flaps. Oral Oncol. 132, 106019. 10.1016/j.oraloncology.2022.106019 35841704

[B14] Mody M. D. Rocco J. W. Yom S. S. Haddad R. I. Saba N. F. (2021). Head and neck cancer. Lancet 398, 2289–2299. 10.1016/s0140-6736(21)01550-6 34562395

[B15] Moratin J. Horn D. Heinemann M. Metzger K. Mrosek J. Ristow O. (2021). Multiple sequential free flap reconstructions of the head and neck: a single-center experience. Plast. Reconstr. Surg. 148, 791e–799e. 10.1097/prs.0000000000008432 34586092

[B16] Qing L. Wu P. Liang J. Yu F. Wang C. Tang J. (2015). Use of flow-through anterolateral thigh perforator flaps in reconstruction of complex extremity defects. J. Reconstr. Microsurg 31, 571–578. 10.1055/s-0035-1555138 26220433

[B17] Raghuram A. C. Manfro G. Teixeira G. V. Cernea C. R. Dias F. L. Marco M. (2021). Use of single chimeric free flaps or double free flaps for complex head and neck reconstruction. J. Reconstr. Microsurg 37, 791–798. 10.1055/s-0041-1727188 33853130

[B18] Ranganath K. Jalisi S. M. Naples J. G. Gomez E. D. (2022). Comparing outcomes of radial forearm free flaps and anterolateral thigh free flaps in oral cavity reconstruction: a systematic review and meta-analysis. Oral Oncol. 135, 106214. 10.1016/j.oraloncology.2022.106214 36302325

[B19] Ritschl L. M. Mucke T. Hart D. Unterhuber T. Kehl V. Wolff K. D. (2021). Retrospective analysis of complications in 190 mandibular resections and simultaneous reconstructions with free fibula flap, iliac crest flap or reconstruction plate: a comparative single centre study. Clin. Oral Investig. 25, 2905–2914. 10.1007/s00784-020-03607-8 PMC8060197 33025147

[B20] Santilli M. D'Addazio G. Rexhepi I. Sinjari B. Filippini A. (2021). Multiple free flap reconstruction of a complex intraoral defect after squamous cell carcinoma excision: a case report. Med. Kaunas. 58, 54. 10.3390/medicina58010054 PMC8781932 35056362

[B21] Schepers R. H. Raghoebar G. M. Vissink A. Stenekes M. W. Kraeima J. Roodenburg J. L. (2015). Accuracy of fibula reconstruction using patient-specific CAD/CAM reconstruction plates and dental implants: a new modality for functional reconstruction of mandibular defects. J. Craniomaxillofac Surg. 43, 649–657. 10.1016/j.jcms.2015.03.015 25911122

[B22] Seruya M. Fisher M. Rodriguez E. D. (2013). Computer-assisted versus conventional free fibula flap technique for craniofacial reconstruction: an outcomes comparison. Plast. Reconstr. Surg. 132, 1219–1228. 10.1097/prs.0b013e3182a3c0b1 23924648

[B23] Shaw R. J. Kanatas A. N. Lowe D. Brown J. S. Rogers S. N. Vaughan E. D. (2004). Comparison of miniplates and reconstruction plates in mandibular reconstruction. Head. Neck 26, 456–463. 10.1002/hed.10343 15122663

[B24] Silva A. K. Humphries L. S. Maldonado A. A. Gottlieb L. J. (2019). Chimeric vs composite flaps for mandible reconstruction. Head. Neck 41, 1597–1604. 10.1002/hed.25606 30775819

[B25] Sozzi D. Novelli G. Silva R. Connelly S. T. Tartaglia G. M. (2017). Implant rehabilitation in fibula-free flap reconstruction: a retrospective study of cases at 1-18 years following surgery. J. Craniomaxillofac Surg. 45, 1655–1661. 10.1016/j.jcms.2017.06.021 28823690

[B26] Taylor G. I. Corlett R. J. Ashton M. W. (2016). The evolution of free vascularized bone transfer: a 40-year experience. Plast. Reconstr. Surg. 137, 1292–1305. 10.1097/prs.0000000000002040 27018684

[B27] Tessler O. Gilardino M. S. Bartow M. J. St Hilaire H. Womac D. Dionisopoulos T. (2017). Transverse cervical artery: consistent anatomical landmarks and clinical experience with its use as a recipient artery in complex head and neck reconstruction. Plast. Reconstr. Surg. 139, 745e–751e. 10.1097/prs.0000000000003085 28234854

[B28] Tharakan T. Marfowaa G. Akakpo K. Jackson R. Zenga J. Puram S. V. (2023). Multiple simultaneous free flaps for head and neck reconstruction: a multi-institutional cohort. Oral Oncol. 136, 106269. 10.1016/j.oraloncology.2022.106269 36462329 PMC10559876

[B29] Thomas W. W. Calcagno H. E. Azzi J. Petrisor D. Cave T. Barber B. (2020). Incidence of inadequate perforators and salvage options for the anterior lateral thigh free flap. Laryngoscope 130, 343–346. 10.1002/lary.28176 31271453

[B30] Townley W. A. Royston E. C. Karmiris N. Crick A. Dunn R. L. (2011). Critical assessment of the anterolateral thigh flap donor site. J. Plast. Reconstr. Aesthet. Surg. 64, 1621–1626. 10.1016/j.bjps.2011.07.015 21840779

[B31] Wang L. Ma C. Y. Shen Y. Fang J. Haugen T. W. Guo B. (2021). Transverse cervical artery anterior perforator flap for head and neck oncological reconstruction: preliminary study. Head. Neck 43, 3598–3607. 10.1002/hed.26873 34510610

[B32] Weitz J. Kreutzer K. Bauer F. J. Wolff K. D. Nobis C. P. Kesting M. R. (2015). Sandwich flaps as a feasible solution for the management of huge mandibular composite tissue defects. J. Craniomaxillofac Surg. 43, 1769–1775. 10.1016/j.jcms.2015.07.038 26330301

[B33] Wu P. Hu L. Li H. Feng L. Liu Y. Zhang S. (2021). Clinical application and accuracy analysis of 3D printing guide plate based on polylactic acid in mandible reconstruction with fibula flap. Ann. Transl. Med. 9, 460. 10.21037/atm-20-6781 33850857 PMC8039666

